# Evaluation of *Meloidogyne chitwoodi* Race 1 and Pathotype Roza in *Arabidopsis thaliana* and Tomato Plants

**DOI:** 10.2478/jofnem-2025-0061

**Published:** 2026-01-14

**Authors:** M. Teixeira, K. S. Clements, K. O. Chandler, C. Gleason

**Affiliations:** Department of Plant Pathology, Washington State University, Pullman, WA 99164

**Keywords:** Columbia root-knot nematode, *Meloidogyne chitwoodi*, Mi-1.2 gene, reproductive factor, Resistance

## Abstract

*Meloidogyne chitwoodi*, commonly known as the Columbia root-knot nematode (CRKN), is a major threat to potato production in the Pacific Northwest (Idaho, Oregon, and Washington) of the United States. The nematode damages both roots and tubers, significantly lowering tuber market value. In Washington and Oregon’s Columbia Basin, the two main *M. chitwoodi* populations are race 1 and a pathotype of race 1 known as Roza. While resistance to race 1 is present in the potato breeding line PA99N82-4, this resistance is ineffective against Roza. To assess virulence differences, both populations were tested on *Arabidopsis thaliana* and tomato roots. Results showed that Roza was more virulent on these two susceptible hosts. Furthermore, infection assays on tomatoes carrying the *Mi-1.2* resistance gene revealed that both populations can overcome this resistance, indicating that *Mi-1.2* does not confer protection against either *M. chitwoodi* race 1 or Roza.

*Meloidogyne chitwoodi* Golden, O’Bannon, Santo, and Finley, commonly called the Columbia root-knot nematode (CRKN), is a major pest of potatoes (*Solanum tuberosum*) (Elling, 2013). Due to its ability to infect potato tubers, *M. chitwoodi* causes internal and external symptoms that render the potatoes unsuitable for fresh markets or processing (Mojtahedi et al., 1991). The nematode has a patchy global distribution, with notable prevalence in the western United States. Its presence in the three primary potato-producing states, Washington, Oregon, and Idaho, poses a substantial challenge for potato growers. The detection of a single juvenile nematode in 250 cm^3^ of soil at planting can lead to economically damaging infestations by harvest (Ingham et al., 2000).

At least three distinct populations of CRKN have been identified, varying in both host range and virulence on resistant potato cultivars (Bali et al., 2021). Races 1 and 2 are separated based on their host range. Race 1 can reproduce on ‘Chantenay’ *carrot* (Daucus carota subsp. *sativus)* but not ‘Thor’ alfalfa (*Medicago sativa*) (Mojtahedi et al., 1988a, 2007; Ingham et al., 2000). Race 2 can reproduce on ‘Thor’ alfalfa but not ‘Chantenay’ carrot (Mojtahedi et al., 1988a, 1988b).

Resistance to *M. chitwoodi* races 1 and 2 was identified in the wild potato species *Solanum bulbocastanum* (Brown et al., 1995; Zhang et al., 2007). The resistance locus from *S. bulbocastanum* was introgressed into cultivated potato, resulting in the resistant breeding line PA99N82-4 (Mojtahedi et al., 1995). The resistance seen in PA99N82-4 is a single dominant gene that is linked to the R*_MC1(blb)_* locus (Brown et al., 2009). Upon CRKN infection of PA99N82-4, avirulent nematodes cause localized cell death responses, called the hypersensitive response (HR), near the head of the nematode. The HR occurs approximately 5 d post-inoculation, thereby preventing the establishment of successful feeding sites (Davies et al., 2014; Bali et al., 2019). This resistance mechanism resembles the resistance conferred by the *Mi-1.2* gene in tomato (*Solanum lycopersicum*), which protects tomatoes against *M. incognita* Kofoid & White, 1919 – Chitwood, 1949, *M. arenaria* Neal, 1889 – Chitwood, 1949, and *M. javanica* – Treub, 1885 – Chitwood, 1949 (Dropkin et al., 1969; Williamson, 1998; Davies et al., 2014).

A population of *M. chitwoodi* capable of overcoming R*_MC1(blb)_*-mediated resistance was identified by Mojtahedi et al. (2007) in field plots. This population, termed Roza, shares the host range of race 1 (i.e., able to reproduce on ‘Chantenay’ carrot but not ‘Thor’ alfalfa) but is virulent on PA99N82-4. Thus, Roza is considered a distinct pathotype of race 1. Genomic sequencing of race 1, race 2, and Roza has enabled the development of diagnostic molecular markers to differentiate these populations (Hu et al., 2023). A regional survey of the potato growing region in the Columbia Basin of Washington and Oregon using these markers found that race 1 and Roza were the two most predominant populations (Hu et al., 2023). Interestingly, several *M. chitwoodi*-positive samples that were confirmed as *M. chitwoodi* via species-specific polymerase chain reaction (PCR) could not be classified using current population-specific primers, suggesting the existence of additional, yet uncharacterized, *M. chitwoodi* populations (Hu et al., 2023).

Despite the genome sequencing and annotation of the three *M. chitwoodi* populations (race 1, race 2, and Roza) (Bali et al., 2021; Teixeira et al., 2025), the genetic determinants underlying their differential host ranges and virulence profiles remain unknown. The primary phenotypic distinction between race 1 and Roza is the latter’s ability to infect potatoes carrying R*_MC1(blb)._* According to Flor’s gene-for-gene model, a dominant plant resistance gene recognizes a specific nematode avirulence (Avr) protein, triggering a HR (Flor, 1971). Roza’s virulence may stem from the loss or mutation of such Avr protein(s), thereby evading resistance recognition. In other plant-pathogen systems, the loss of Avr genes has been associated with reduced pathogen virulence or overall fitness (Dutta et al., 2021). For example, in fungal pathogens, there is evidence that loss of Avr proteins can result in reduced virulence and aggressiveness (Pariaud et al., 2009), and similarly for bacterial pathogens, the loss of Avr-proteins can lead to a loss of fitness on susceptible hosts (Laine and Barrès, 2013).

Although race 1 and Roza exhibit no apparent morphological differences, their virulence profiles on PA99N82-4 are different (Mojtahedi et al., 1994; Hu et al., 2023). The resistance in PA99N82-4 is believed to be governed by a single dominant locus R*_MC1(blb)_* (Brown et al., 2009). The resistance-breaking Roza population is morphologically similar to race 1, and the ability of Roza to break R*_MC1(blb)_*-mediated resistance suggests that this population underwent adaptations enabling it to bypass effector-triggered immunity (Davies et al., 2014). We hypothesized that Roza may incur a fitness cost as a resistance-breaking population. To test this, we assessed the virulence of both populations on wild-type *A. thaliana* and ‘Rutgers’ tomato, as well as on a tomato harboring the *Mi-1.2* resistance gene (‘Better Boy); tomatoes carrying the *Mi-1.2* gene had previously demonstrated efficacy in suppressing race 1 reproduction (Brown et al., 1997).

## Materials and Methods

### Nematode cultures

The *M. chitwoodi* race 1 and Roza egg inoculums were initially provided by Dr. Charles Brown (USDA-ARS), and populations were confirmed by population-specific PCR (Table 1) (Hu et al., 2023). The *M. incognita* population was provided by Dr. Inga Zasada (USDA-ARS). All nematode populations were maintained in a greenhouse, on susceptible ‘Rutgers’ tomatoes. To obtain nematode eggs from inoculated tomato plants, tomato roots were shaken in a 0.6% sodium hypochlorite solution for 3 min. After passing the solution through a set of sieves (pore sizes of 125 µm, 45 µm, and 25 µm), the nematode eggs were collected on the 25 µm sieve and subjected to sucrose floatation (Jacob and van Bezooijen, 1984).

**Table 1:** Primers used in the experiments.

	**Primer**	**Sequence 5′ to 3′**	**Reference**
Mi genotyping	Mi23F	TGGAAAAATGTTGAATTTCTTTTG	Seah et al. (2007)
	Mi23R	GCATACTATATGGCTTGTTTACCC	
*M. chitwoodi* race1	Mc1MR_F	TTTTGTGCGATTGGCAAGGA	Hu et al. (2023)
	Mc1MR_R	ATACATTCGCTCGGTTCCCG	
*M. chitwoodi* Roza	ROZMR_F	CACGGAACGTTGGAAGGGTA	Hu et al. (2023)
	ROZMR_R	CCTCGTGATCAGCGCAGTAA	

To hatch the eggs and collect infective second-stage juveniles (J2), the eggs were surface sterilized using a 10% household bleach (6% hypochlorite diluted to 0.6% final) solution for 3 min, followed by rinsing with sterile water three times. Then, the eggs were resuspended in 0.1% plant preservative mixture (Plant Cell Technology, Washington DC) and incubated in a modified Baermann beaker in the dark at room temperature (Zhang and Gleason, 2021). J2s were collected after 2 d of incubation in the Baermann beaker.

### Inoculation of Arabidopsis seedlings

Wild-type *A. thaliana* Col-0 seeds were surface sterilized using 75% ethanol for 5 min and dried inside a laminar flow hood for 20 min followed by cold treatment in the dark at 4°C for 3 d. The seeds were placed on Murashige and Skoog (MS) media (4.3 g/l MS basal salts with micronutrients and vitamins), sucrose (30 g/l), and Gelzan (4 g/l), with a pH of 5.8–6.0 and incubated in a growth chamber set to standard conditions (23°C, 12 hr:12 hr light:dark cycle).

One week later, seedlings were transferred to infection media (MS salts [4.3 g/l], sucrose [10 g/l], and Gelzan [8 g/l], pH 5.7) with 3 seedlings/plate, and returned to incubation in the growth chamber. When the seedlings were 2-wk-old, they were inoculated with approximately 150 freshly hatched J2s per plant, and the plants were incubated in the growth chamber at standard conditions. Seedlings were evaluated 2 wk post-inoculation for root galling and root weights.

### Inoculation of tomato plants

Tomato seeds from the cultivars ‘Rutgers’ (*mi/mi*) and ‘Better Boy’ (*Mi/mi*) were germinated in 1-l pots of sand in a greenhouse (23°C, 14 hr light:10 hr dark). Once the seedlings had two fully expanded leaves, they were transplanted to individual 21.6 cm × 3.8 cm cone-tainers (Steuwe and Sons, Inc., True Seedling Nursery Containers, Tangent, OR) filled with sand. Four-week-old seedlings were inoculated with eggs. For the egg mass assay, each plant was inoculated with 3,000 eggs, and eggs and galls were counted at 30 d post-inoculation (dpi). There were 15–18 ‘Rutgers’ (*mi/mi*) and 15–18 ‘Better Boy’ (*Mi/mi*) tested for each experiment, and experiments were repeated at least twice. For measuring the reproductive factor (RF) values, the protocol for measuring *M. chitwoodi* and *M. incognita* RF on *Mi-1.2* tomatoes described in Brown et al. (1997) was followed; 5,000 eggs per plant were used as the inoculum, and eggs were collected at 60 dpi (Brown et al., 1997). There were 9–7 ‘Rutgers’ (*mi/mi*) and 7–10 ‘Better Boy’ used for each independent experiment, which was performed twice.

For the evaluation of egg masses, plants were removed from the cone-tainers and gently rinsed. The roots were weighed and then stained using Ponceau coloring to visualize egg masses (Thies et al., 2002). For the RF evaluation, plants were removed from the cone-tainers, and eggs were extracted from individual root systems using the method described for egg collection for inoculation. RF (RF = final population [Pf]/initial population [Pi]).

All the counting for J2s, egg, egg masses, and galls were performed using a Zeiss Stemi 2000C Stereo Microscope manufactured by Zeiss. Data was subjected to Mann-Whitney test or Tukey's multiple comparison test using GraphPad Prism software version 10.0.0 for Windows, manufactured by GraphPad Software, Boston, Massachusetts USA, www.graphpad.com .

### DNA extraction and PCR

PCR was performed to confirm the genotypes of the nematodes. Gall samples were collected after evaluation of the RF values and subjected to DNA extraction using a method adapted from Edwards et al. (1991). Briefly, large galls from the infected tomatoes were cut from the roots and quickly frozen in liquid nitrogen. The frozen galls were ground with a small pestle in a 1.7 ml Eppendorf (Hamburg, Germany) tube, and DNA was extracted using 400 µl extraction buffer (200 mM Tris-HCL pH 7.5, 250 mM NaCl, 25 mM ethylenediaminetetraacetic acid, and 0.5% SDS) and vortexed for 5 sec. The extracts were centrifuged at 13,000 rpm in an Eppendorf centrifuge 5424 with a rotor diameter of 11 cm for 1 min, and 300 µl of the supernatant was transferred to a fresh Eppendorf tube. This supernatant was mixed with 300 µl isopropanol and left at room temperature for 2 min. Following centrifugation at 13,000 rpm for 5 min, the pellet was air dried and dissolved in 100 µl H_2_O. The resulting DNA was a mixture of nematode and tomato. Approximately 2–10 ng DNA was used to perform PCR to confirm the genotypes of the nematodes (Hu et al., 2023) and the *Mi* + tomato (Seah et al., 2007) using the primers in Table 1.

## Results

### The *M. chitwoodi* Roza population is more virulent on Arabidopsis and tomato compared to race 1

**Figure 1:**
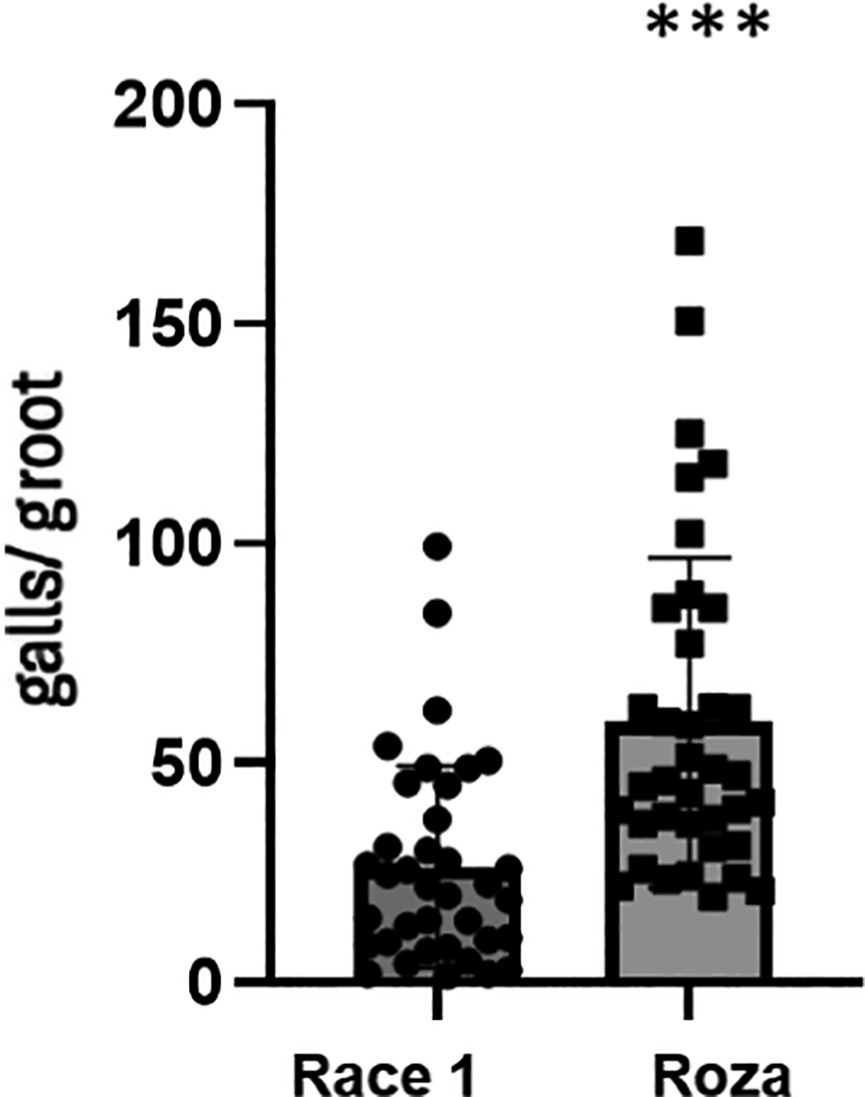
*Meloidogyne chitwoodi* Roza causes more galls than race 1 on Arabidopsis roots. 2-wk-old *Arabidopsis* seedlings were inoculated with approximately 150 J2s of *M. chitwoodi* race 1 or Roza. Galls per gram (g) of roots were counted at 14 d post-inoculation. Data represents the mean galls per gram of plant roots for two independent experiments combined (n = 37) +/− SD ^***^P-value <0.0001, Mann–Whitney test. SD, standard deviation.

To investigate the virulence of the two *M. chitwoodi* populations, race 1 and Roza, on wild-type *A. thaliana* (Col-0) plants, 2-wk-old seedlings were inoculated with 150 J2s per plant. At 2-wk-old post-inoculation, the roots were evaluated for galling. Roza caused significantly more gall formation per gram of root than race 1 on the *A. thaliana* roots (Figure 1).

**Figure 2:**
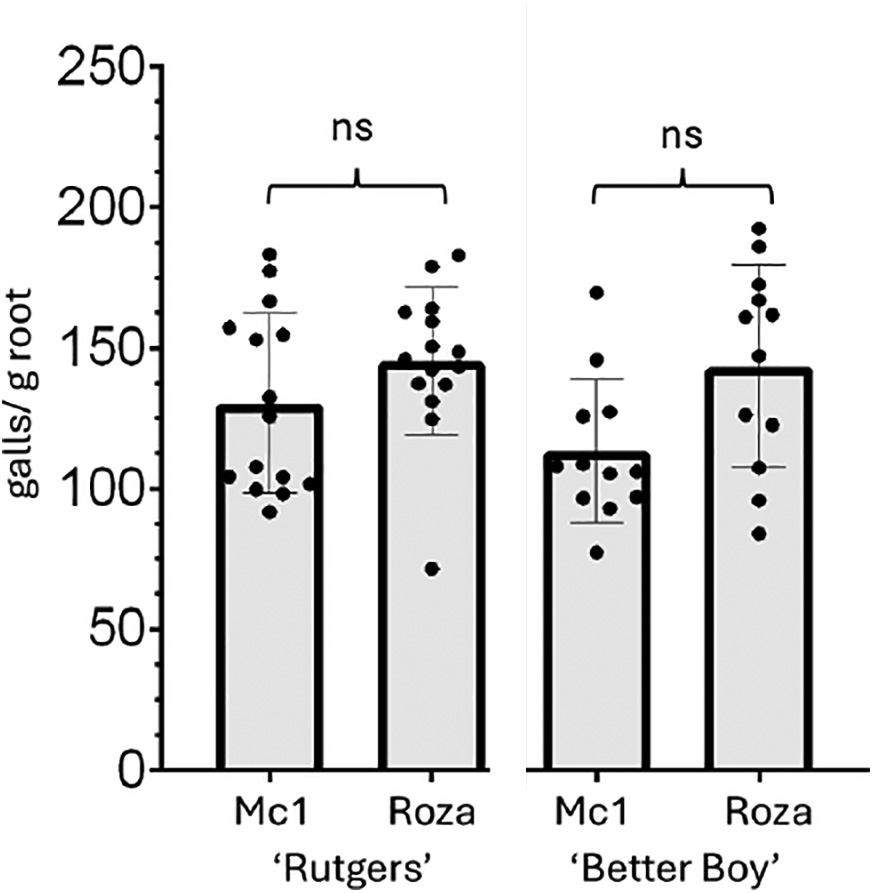
*Meloidogyne chitwoodi* race 1 and Roza can infect ‘Rutgers’ (*mi/mi*) and ‘Better Boy’ (*Mi/mi*). 4-wk-old tomato plants were inoculated with 3,000 eggs of either race 1 or Roza. Galling severity was quantified as the number of galls per gram (g) of root at 30 dpi. Bars represent mean galls per gram of root (*n* = 12–15 plants) ± SD, and experiment repeated twice with similar results. ns, as determined by Mann–Whitney test. ns, not significant; SD, standard deviation.

The susceptibility of the tomato cultivar ‘Rutgers’, which lacks the nematode resistance gene *Mi-1.2*, was evaluated against race 1 and Roza. ‘Rutgers’, tomato seedlings were inoculated with approximately 3,000 eggs per plant from either race 1 or Roza, and gall formation was assessed 30 dpi. Galling was observed on ‘Rutgers’ plants inoculated with both race 1 and Roza (Figure 2). There was a trend toward more galling in ‘Rutgers’ infected with Roza compared to ‘Rutgers’ infected with race 1.

Then, the ability of race 1 and Roza to infect and form galls on the resistant tomato cultivar ‘Better Boy’ (*Mi/mi*) was tested. ‘Better Boy’ (*Mi/mi*) was inoculated with 3,000 eggs, and the number of galls per root system was counted at 30 dpi. Both race 1 and Roza successfully infected ‘Better Boy’ and induced gall formation (Figure 2). There was a trend toward more galling on ‘Better Boy’ infected with Roza compared with race 1.

### ‘Better Boy’ tomato (+*Mi*) is susceptible to *M. chitwoodi* race 1 and Roza

**Figure 3:**
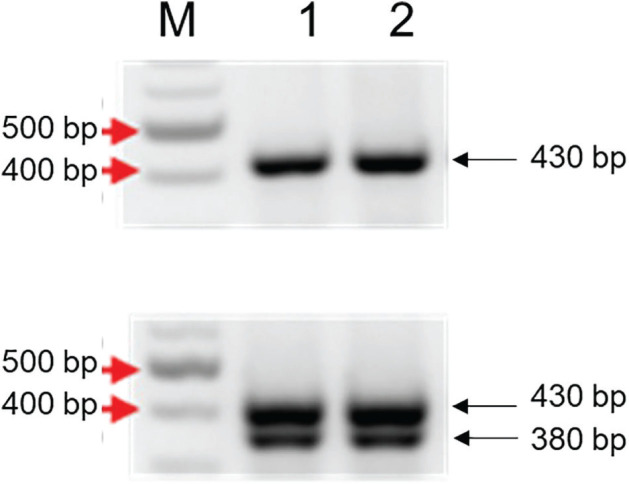
Detection of *Mi-1.2* in ‘Better Boy’ tomatoes. Galls were collected from *M. chitwoodi-*infected ‘Better Boy’ and ‘Rutgers’ tomatoes and subjected to DNA extraction and PCR with primers Mi23F and Mi23R (Seah et al., 2007). PCR from ‘Rutgers’ results in a 430 bp band, indicating that it is the *mi1.2* susceptible allele. PCR from ‘Better Boy’ resulted in 380 bp and 430 bp bands, indicating that the plant is heterozygous for *Mi1.2* resistance. 1 Kb + ladder, from New England Biolabs. Red arrows indicate band sizes. Lanes 1 and 2 indicate that PCR was performed on DNA from nematodes inside galls from two independent plants. MM, molecular marker; PCR, polymerase chain reaction.

**Figure 4:**
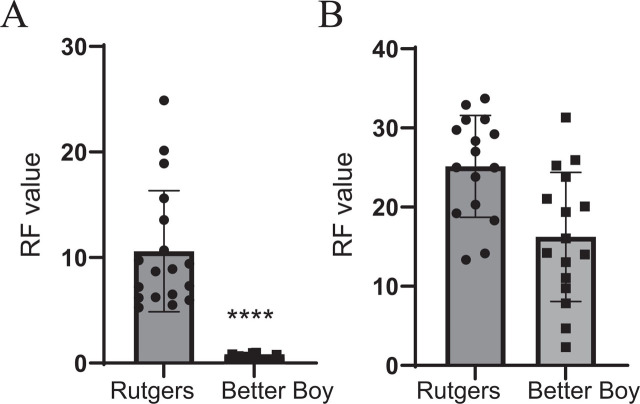
RF values for *M. incognita* and *M. chitwoodi* race 1 on ‘Rutgers’ and ‘Better Boy’. (A) 4-wk-old tomato plants were inoculated with 5,000 *M. incognita* eggs and evaluated 60 d later for RF values. Data represent the mean reproduction factor per plant of two independent experiments combined (*n* = 18–19) ± SD. (B) 4-wk-old tomato plants were inoculated with 5,000 *M. chitwoodi* race 1 eggs and evaluated 60 d later for RF values. *n* = 16. Data represent the mean reproduction factor per plant of two independent experiments combined (*n* = 16) ± SD. Statistical significance was determined using the Mann–Whitney test ^*^P < 0.002; ^***^P < 0.001. RF, reproductive factor; SD, standard deviation.

A previous study found that tomatoes carrying the resistance gene *Mi-1.2* were resistant to race 1 (Brown et al., 1997), but Roza had never been evaluated on *Mi*-containing tomatoes. Since ‘Better Boy’ was used as the resistant control in subsequent experiments, PCR with *Mi-1.2*-specific primers was performed to verify the presence of the gene (Figure 3). Infection assays with *M. incognita* showed that the RF value was less than one in ‘Better Boy’ (*Mi/mi*) roots (Figure 4). There is significantly less *M. incognita* reproduction on ‘Better Boy’ than on ‘Rutgers’. These data confirmed that ‘Better Boy’ is a poor host (i.e., resistant) to *M. incognita* (Ferris et al., 1993).

A previous report showed that tomatoes carrying the *Mi-1.2* gene were resistant to race 1, with an RF < 1 (Brown et al., 1997), but our data showed that race 1 can infect and produce galls in ‘Better Boy’ tomatoes, which contain the *Mi-1.2* gene. The RF for race 1 was assessed on ‘Better Boy’ and ‘Rutgers’ roots. ‘Better Boy’ plants had an RF > 1, like the susceptible control ‘Rutgers’ (Figure 3).

## Discussion

Previous studies have reported that Roza is more aggressive than race 1 on both a susceptible potato cultivar and on the susceptible ‘Rutgers’ tomatoes (Brown et al., 2009). In this study, we confirmed that Roza exhibits increased aggressiveness, not only on ‘Rutgers’ tomatoes, but also on the model plant *A. thaliana.* This observation suggests that the acquisition of virulence against resistant potato genotypes did not compromise its fitness on susceptible hosts and that the cost of virulence may be minimal in this population (Dutta et al., 2021). Although PA99N82-4 is not commercially deployed in potato production, Roza is now widespread throughout Washington (Hu et al., 2023). Its broad distribution may be attributed to its increased aggressiveness and potential lack of fitness penalties associated with virulence.

Genetic resistance against root-knot nematodes is well characterized in tomato with the *Mi-1.2* gene (Milligan et al., 1998). Despite emerging reports of resistance-breaking nematode populations, *Mi-1.2* remains widely used to manage *M. incognita*, *M. javanica*, and *M. arenaria* in tomato (Ploeg et al., 2023). The longstanding success of *Mi-1.2* in tomato for controlling root-knot nematodes has prompted interest in its transfer to other crops. It has been successfully expressed in eggplant and lettuce, conferring resistance to the same three root-knot nematode species (Goggin et al., 2006; Zhang et al., 2010). However, attempts to express the gene in *A. thaliana* and *Nicotiana benthamiana* have failed to trigger root-knot nematode resistance, suggesting that some plant species may lack the necessary downstream signaling components required for *Mi-1.2*-mediated defenses (Goggin et al., 2006).

Given the absence of genetic resistance to *M. chitwoodi* in cultivated potato, one proposed strategy against *M. chitwoodi* would be the introduction of the *Mi-1.2* gene from tomato into potato. However, before this gene can be transferred, its effectiveness against *M. chitwoodi* populations must first be demonstrated in tomato. In a study by Brown et al. (1997), *Mi-1.2* tomatoes were evaluated against four *M. chitwoodi* populations (Brown et al., 1997). The *Mi-1.2* gene conferred resistance to race 1 and two isolates of race 2, reducing nematode reproduction. However, it did not provide resistance to race 3 or to *M. hapla* (Brown et al., 1997). Race 3, also known as CAMC2, was first identified in Tulelake, California (Mojtahedi et al., 1994). It is considered a virulent variant of race 2, capable of reproducing on both ‘Thor’ alfalfa and *S. bulbocastanum*, the source of resistance in PA99N82-4 (Mojtahedi et al., 1994; Brown et al., 2009). The authors concluded that *Mi-1.2* may provide useful resistance to *M. chitwoodi* race 1 if successfully transferred into potato. However, the gene’s effectiveness against Roza was not evaluated.

Following our confirmation that Roza is an aggressive race 1 pathotype, we sought to determine whether a tomato hybrid carrying the *Mi-1.2* gene could resist this population. To test this, we used ‘Better Boy’ tomatoes, which is heterozygous for the *Mi-1.2* gene (Figure 3) and exhibits resistance to *M. incognita*. Since *Mi-1.2* is a single dominant gene (El-Sappah et al., 2019), nematode resistance is retained in heterozygous (*Mi/mi*) plants. Inoculation of ‘Better Boy’ with Roza revealed that this population can overcome *Mi-1.2*-mediated resistance. Although there was a trend toward fewer galls compared to the susceptible ‘Rutgers’ tomatoes, galling still occurred, and there was no statistical difference in the number of galls produced by Roza on ‘Better Boy’ and ‘Rutgers’.

**Figure 5:**
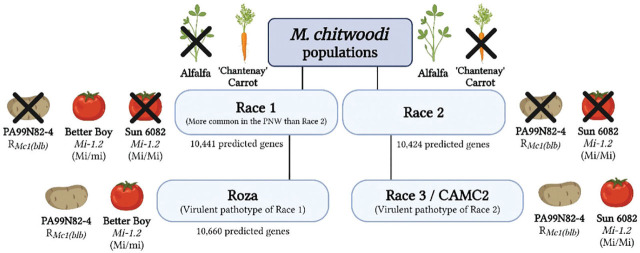
Diagram showing populations of *M. chitwoodi*, with black X’s marking plants where the nematodes do not reproduce. Races 1 and 2 are distinguished by their ability to reproduce either on ‘Thor’ alfalfa or ‘Chantenay’ carrot due to non-host immunity (Mojtahedi et al., 1988). In this study, race 1 is shown to infect ‘Better Boy’ tomato (*Mi*/*mi*). Previous research found that both races 1 and 2 cannot reproduce on Sun 6082 tomato (*Mi/Mi*) or PA99N82-4 potato carrying R*_MC1(blb)_* resistance (Brown et al., 1997, 2009). Race 3, a virulent pathotype of race 2, can infect and reproduce on both PA99N82-4 and Sun 6082 (*Mi/Mi*). The Roza population can also infect and reproduce on PA99N82-4, though it has not been tested on Sun 6082. This study shows that Roza can infect and reproduce on ‘Better Boy’ (*Mi/mi*), suggesting that *Mi-1.2* resistance cannot control this *M. chitwoodi* population. The number of genes associated with race 1, Roza, and race 2 was estimated by Bali et al. (2021).

The race 1 population also overcame *Mi-1.2* resistance in ‘Better Boy’, which contradicts a previous report that *Mi-1.2* confers resistance to race 1 (Brown et al., 1997). In the Brown et al., 1997 paper, the RF value of race 1 on *Mi-1.2* tomato Sun 6082 was significantly lower than on susceptible control Castlerock *(mi*/*mi),* suggesting that race 1 is avirulent on tomatoes containing *Mi-1.2*. There may be several explanations for the difference in race 1 virulence on *Mi*+−tomatoes. The race 1 population used in our study and in Brown et al. (2007) originated from the same source, but it is possible that maintenance on ‘Rutgers’ tomato since 2016 at Washington State University has led to genetic shifts or adaptations. Additionally, the *Mi* + −tomato used in the Brown et al. (1997) study was Sun 6082 *(Mi*/*Mi),* which differs from the ‘Better Boy’ hybrid *(Mi*/*mi)* used in this study. The Sun 6082 tomato line is a near-isogenic relative of the susceptible line Castlerock *(mi*/*mi).* Based on the higher RF values for race 1 in the susceptible tomato control Columbia (*mi/mi*) compared to Castlerock (*mi/mi*), the authors speculated that Castlerock and Sun 6082 may carry additional nematode resistance genes (Brown et al., 1997). Therefore, the low RF values observed in *Mi*+ −tomatoes (Sun 6082) infected with race 1 by Brown et al. (1997) may be due to the presence of these additional resistance factors working in combination with *Mi-1.2*. Our data show that Roza is more aggressive than race 1 on Arabidopsis and tomato. Moreover, *Mi-1.2*-mediated resistance in tomato is not effective against two *M. chitwoodi* populations. Current available information about the different *M. chitwoodi* populations is consolidated in Figure 5, which shows that race 1 can overcome *Mi-1.2* (*Mi*/*mi*) resistance in ‘Better Boy’ tomato. The figure also summarizes the findings from Brown et al. (1997), which found that both races 1 and 2 are unable to overcome resistance in Sun 6082 tomato (*Mi*/*Mi*) or the R*_MC1(blb)_* resistance in PA99N82-4 potato (Brown et al., 1997, 2009). Race 3, a virulent pathotype of race 2, can infect both PA99N82-4 and Sun 6082 (*Mi*/*Mi*). The Roza population can also infect PA99N82-4. While Roza has not been tested on Sun 6082, this study shows that it can overcome *Mi-1.2* resistance in ‘Better Boy’ (*Mi*/*mi*). Future studies should explore a broader host range for all four populations, as well as investigate potential fitness costs associated with virulence. Overall, the lack of effective resistance conferred by *Mi-1.2* in ‘Better Boy’ against both race 1 and Roza casts doubt on the utility of this gene for protecting potato from *M. chitwoodi.*

## References

[j_jofnem-2025-0061_ref_001] Bali S., Hu S., Vining K., Brown C., Mojtahedi H., Zhang L., Gleason C., Sathuvalli V. (2021). Nematode genome announcement: Draft genome of *Meloidogyne chitwoodi*, an economically important pest of potato in the Pacific Northwest. Molecular Plant-Microbe Interactions.

[j_jofnem-2025-0061_ref_002] Bali S., Vining K., Gleason C., Majtahedi H., Brown C. R., Sathuvalli V. (2019). Transcriptome profiling of resistance response to *Meloidogyne chitwoodi* introgressed from wild species *Solanum bulbocastanum* into cultivated potato. BMC Genomics.

[j_jofnem-2025-0061_ref_003] Brown C. R., Mojtahedi H., Santo G. S. (1995). Introgression of resistance to Columbia and northern root-knot nematodes from *Solanum bulbocastanum* into cultivated potato. Euphytica; Netherlands Journal of Plant Breeding.

[j_jofnem-2025-0061_ref_004] Brown C. R., Mojtahedi H., Santo G. S., Williamson V. M. (1997). Effect of the *Mi* gene in tomato on reproductive factors of *Meloidogyne chitwoodi* and *M. hapla*. Journal of Nematology.

[j_jofnem-2025-0061_ref_005] Brown C. R., Mojtahedi H., Zhang L. H., Riga E. (2009). Independent resistant reactions expressed in root and tuber of potato breeding lines with introgressed resistance to *Meloidogyne chitwoodi*. Phytopathology.

[j_jofnem-2025-0061_ref_006] Davies L., Brown C., Elling A. (2014). Calcium is involved in the R Mc1 (blb)-mediated hypersensitive response against *Meloidogyne chitwoodi* in potato. The Plant Cell Reports.

[j_jofnem-2025-0061_ref_007] Dropkin V. H., Helgeson J. P., Upper C. D. (1969). The hypersensitivity reaction of tomatoes resistant to *Meloidogyne incognita*: Reversal by cytokinins. Journal of Nematology.

[j_jofnem-2025-0061_ref_008] Dutta A., Croll D., McDonald B. A., Barrett L. G. (2021). Maintenance of variation in virulence and reproduction in populations of an agricultural plant pathogen. Evolutionary Applications.

[j_jofnem-2025-0061_ref_009] Edwards K., Johnstone C., Thompson C. (1991). A simple and rapid method for the preparation of plant genomic DNA for PCR analysis. Nucleic Acids Research.

[j_jofnem-2025-0061_ref_010] Elling A. A. (2013). Major emerging problems with minor meloidogyne species. Phytopathology.

[j_jofnem-2025-0061_ref_011] El-Sappah A. H., Islam M. M., El-Awady H. H., Yan S., Qi S., Liu J., Cheng G. T., Liang Y. (2019). Tomato natural resistance genes in controlling the root-knot nematode. Genes (Basel).

[j_jofnem-2025-0061_ref_012] Ferris H., Carlson H. L., Viglierchio D. R., Westerdahl B. B., Wu F. W., Anderson C. E., Juurma A., Kirby D. W. (1993). Host status of selected crops to *Meloidogyne chitwoodi*. Journal of Nematology.

[j_jofnem-2025-0061_ref_013] Flor H. (1971). Current status of the genefor-gene concept. Annual Review of Phytopathology.

[j_jofnem-2025-0061_ref_014] Goggin F. L., Jia L., Shah G., Hebert S., Williamson V. M., Ullman D. E. (2006). Heterologous expression of the *Mi-1.2* gene from tomato confers resistance against nematodes but not aphids in rgg-plant. Molecular Plant-Microbe Interactions.

[j_jofnem-2025-0061_ref_015] Hu S., Franco J., Bali S., Chavoshi S., Brown C., Mojtahedi H., Quick R., Cimrhakl L., Ingham R., Gleason C., Sathuvalli V. (2023). Diagnostic molecular markers for identification of different races and a pathotype of Columbia root knot nematode. PhytoFrontiers.

[j_jofnem-2025-0061_ref_016] Ingham R. E., Hamm P. B., Williams R. E., Swanson W. H. (2000). Control of *Meloidogyne chitwoodi* in potato with fumigant and nonfumigant nematicides. Journal of Nematology.

[j_jofnem-2025-0061_ref_017] Jacob J. J., van Bezooijen J. (1984). Manual for practical work in nematology, revised 1984.

[j_jofnem-2025-0061_ref_018] Laine A.-L., Barrès B. (2013). Epidemiological and evolutionary consequences of life-history tradeoffs in pathogens. Plant Pathology.

[j_jofnem-2025-0061_ref_019] Milligan S. B., Bodeau J., Yaghoobi J., Kaloshian I., Zabel P., Williamson V. M. (1998). The root knot nematode resistance gene *Mi* from tomato is a member of the leucine zipper, nucleotide binding, leucine-rich repeat family of plant genes. The Plant Cell.

[j_jofnem-2025-0061_ref_020] Mojtahedi H., Brown C. R., Riga E., Zhang L. H. (2007). A new pathotype of *Meloidogyne chitwoodi* race 1 from Washington State. Plant Disease.

[j_jofnem-2025-0061_ref_021] Mojtahedi H., Brown C., Santo G. (1995). Characterization of resistance in a somatic hybrid of *Solanum bulbocastanum* and *S. tuberosum*, to *Meloidogyne chitwoodi*. Journal of Nematology.

[j_jofnem-2025-0061_ref_022] Mojtahedi H., Ingram R. E., Santo G. S., Pinkerton J. N., Reed G. L., Wilson J. H. (1991). Seasonal migration of *Meloidogyne chitwoodi* and its role in potato production. Journal of Nematology.

[j_jofnem-2025-0061_ref_023] Mojtahedi H., Santo G. S., Brown C. R., Ferris H., Williamson V. M. (1994). A new host race of *Meloidogyne chitwoodi* from California. Plant Disease.

[j_jofnem-2025-0061_ref_024] Mojtahedi H., Santo G. S., Wilson J. H. (1988a). Host tests to differentiate *Meloidogyne chitwoodi* races 1 and 2 and *M. hapla*. Journal of Nematology.

[j_jofnem-2025-0061_ref_025] Mojtahedi H., Santo G. S., Pinkerton J. N. (1988b). Differential response of Thor alfalfa to *Meloidogyne chitwoodi* races and *M. hapla*. Journal of Nematology.

[j_jofnem-2025-0061_ref_026] Pariaud B., Ravigné V., Halkett F., Goyeau H., Carlier J., Lannou C. (2009). Aggressiveness and its role in the adaptation of plant pathogens. Plant Pathology.

[j_jofnem-2025-0061_ref_027] Ploeg A. T., Stoddard C. S., Turini T. A., Nunez J. J., Miyao E. M., Subbotin S. A. (2023). Tomato *Mi*-gene resistance-breaking populations of *Meloidogyne* show variable reproduction on susceptible and resistant crop cultivars. Journal of Nematology.

[j_jofnem-2025-0061_ref_028] Seah S., Telleen A. C., Williamson V. M. (2007). Introgressed and endogenous *Mi-1* gene clusters in tomato differ by complex rearrangements in flanking sequences and show sequence exchange and diversifying selection among homologues. Theoretical and Applied Genetics.

[j_jofnem-2025-0061_ref_029] Teixeira M., Ko I., Bali S., Vieira P., Maier T. R., Baum T. J., Gleason C. (2025). Genome reannotation and effector candidate identification in *Meloidogyne chitwoodi* through gland-specific transcriptome analysis identify new effector candidates in Meloidogyne chitwoodi. PLOS Pathogens, 2025.

[j_jofnem-2025-0061_ref_030] Thies J. A., Merrill S. B., Corley E. L. (2002). Red food coloring stain: New, safer procedures for staining nematodes in roots and egg masses on root surfaces. Journal of Nematology.

[j_jofnem-2025-0061_ref_031] Williamson V. M. (1998). Root-knot nematode resistance genes in tomato and their potential for future use. Annual Review of Phytopathology.

[j_jofnem-2025-0061_ref_032] Zhang L., Gleason C. (2021). Transcriptome analyses of pre-parasitic and parasitic *Meloidogyne chitwoodi* race 1 to identify putative effector genes. Journal of Nematology.

[j_jofnem-2025-0061_ref_033] Zhang L. H., Mojtahedi H., Kuang H., Baker B., Brown C. (2007). Marker-assisted selection of Columbia root-knot nematode resistance introgressed from *Solanum bulbocastanum*. Crop Science.

[j_jofnem-2025-0061_ref_034] Zhang L. Y., Zhang Y. Y., Chen R. G., Zhang J. H., Wang T. T., Li H. X., Ye Z. B. (2010). Ectopic expression of the tomato *Mi-1* gene confers resistance to root knot nematodes in lettuce (*Lactuca sativa*). Plant Molecular Biology Reporter.

